# Reduction in Insulin Mediated ERK Phosphorylation by Palmitate in Liver Cells Is Independent of Fatty Acid Induced ER Stress

**DOI:** 10.3390/nu14173641

**Published:** 2022-09-02

**Authors:** Sindiyan Alshaikh Mubarak, Abeer Al Otaibi, Ali Al Qarni, Ahmed Bakillah, Jahangir Iqbal

**Affiliations:** King Abdullah International Medical Research Center-Eastern Region, King Abdulaziz Hospital, King Saud Bin Abdulaziz University for Health Sciences, Ministry of National Guard-Health Affairs, Al-Ahsa 31982, Saudi Arabia

**Keywords:** insulin resistance, insulin signaling, ER stress, unfolded protein response, palmitate, oleate, tunicamycin

## Abstract

Saturated free fatty acids (FFAs) such as palmitate in the circulation are known to cause endoplasmic reticulum (ER) stress and insulin resistance in peripheral tissues. In addition to protein kinase B (AKT) signaling, extracellular signal-regulated kinase (ERK) has been implicated in the development of insulin resistance. However, there are conflicting data regarding role of ERK signaling in ER stress-induced insulin resistance. In this study, we investigated the effects of ER stress on insulin resistance and ERK phosphorylation in Huh-7 cells and evaluated how oleate prevents palmitate-mediated ER stress. Treatment with insulin resulted in an increase of 38–45% in the uptake of glucose in control cells compared to non-insulin-treated control cells, along with an increase in the phosphorylation of AKT and ERK. We found that treatment with palmitate increased the expression of ER stress genes, including the splicing of *X box binding protein 1* (*XBP1*) mRNA. At the same time, we observed a decrease in insulin-mediated uptake of glucose and ERK phosphorylation in Huh-7 cells, without any change in AKT phosphorylation. Supplementation of oleate along with palmitate mitigated the palmitate-induced ER stress but did not affect insulin-mediated glucose uptake or ERK phosphorylation. The findings of this study suggest that palmitate reduces insulin-mediated ERK phosphorylation in liver cells and this effect is independent of fatty-acid-induced ER stress.

## 1. Introduction

It is well known that elevated levels of plasma saturated free fatty acids (FFAs) cause insulin resistance in the liver and muscles [1]. These FFAs also accumulate in non-adipose tissues and cause deleterious effects such as lipotoxicity [1]. Lipotoxicity is known to contribute to the pathogenesis of obesity-related complications such as metabolic syndrome and diabetes mellitus [2]. Strategies to inhibit lipotoxicity-mediated signaling cascades are important to prevent obesity-related metabolic complications. The fatty acid composition of dietary fats is a critical factor in determining the lipotoxic activities in cells [3]. Saturated fatty acids such as palmitate are mostly cytotoxic, whereas unsaturated fatty acids such as oleate are non-toxic or cytoprotective [4,5,6]. The differential effect of palmitate and oleate on the fate of cells depends on their concentration, the ratio of these fatty acids and the cell type [7,8,9]. Studies have shown that oleate may protect against palmitate-induced cell death by forming neutral lipids, such as triglycerides, which are stored as lipid droplets in the cells [10,11,12,13].

Palmitate induces endoplasmic reticulum (ER) stress and activates an unfolded protein response (UPR) that can be mitigated by oleate [14,15,16]. ER stress refers to an imbalance between the demand for protein folding and the capacity of the ER for protein folding. The UPR serves to increase the fidelity of protein folding by decreasing protein translation, increasing chaperone expression and accelerating the degradation of misfolded proteins via three main effectors—inositol-requiring enzyme-1 alpha (IRE1α), activating transcription factor 6 (ATF6) and protein kinase R-like ER protein kinase (PERK) [1]. Upon activation, these pathways induce the expression of several genes and chaperones, such as CCAAT/enhancer binding protein (C/EBP), homologous protein (CHOP) and BIP/GRP-78, to restore ER homeostasis. The induction of UPR through IRE1α activates the transcription factor *X box binding protein 1* (*XBP1*) by means of an endonucleatic cleavage of a 26 bp segment of its mRNA [17].

Prolonged exposure of liver cells to elevated levels of saturated long-chain fatty acids such as palmitate results in abnormal insulin signaling [18,19]. Insulin resistance in liver cells is one of the clinical features of diabetes mellitus which is associated with impaired signal transduction and glucose transport [20,21,22]. Protein kinase B (AKT) phosphorylation is a downstream effector of insulin signaling that is directly linked to the regulation of glucose transport [23]. In addition to AKT signaling, extracellular signal-regulated kinase (ERK) has been implicated in the development of insulin resistance associated with obesity and diabetes mellitus [24]. However, there are conflicting data regarding role of ERK signaling in ER stress-induced insulin resistance [25,26].

The present study was undertaken to determine the effect of palmitate on insulin-mediated glucose uptake and the related signaling pathway in Huh-7 cells. In this study we also aimed to analyze the underlying mechanisms involved in insulin resistance and ER stress caused by palmitate.

## 2. Materials and Methods

### 2.1. Materials

TRIzol^TM^ (catalog #15596018) was purchased from Life Technologies (Carlsbad, CA, USA). A High-Capacity cDNA reverse transcription kit (catalog #4368813) was purchased from Thermo Fisher Scientific (Waltham, MA, USA). A qPCR^TM^ core kit for SYBR Green I (catalog #10-SN10-05) was purchased from Eurogentec (San Diego, CA, USA). 2-NBD glucose (catalog #7133) was purchased from Setareh Biotech (Eugene, OR, USA). Tunicamycin (catalog #T7765), oleate (catalog #O1008), palmitate (catalog #P0500) and sulfo-*N*-succinimidyl oleate sodium (catalog #SML2148) were purchased from Sigma Aldrich (St. Louis, MO, USA). PERK (catalog #5683), IRE1α (catalog #3294), ATF6 (catalog #65880), BIP (catalog #3177), CHOP (catalog #5554), pAS160 (catalog #8730), pAKT (catalog #4060), pERK (catalog #45899) and ACTIN (catalog #4970) Rabbit mABs were purchased Cell Signaling (Danvers, MA, USA). HRP anti-rabbit secondary IgG antibodies (catalog #ab288151) were purchased from Abcam (Cambridge, MA, USA). All other chemicals and solvents were obtained from Fisher Scientific through its local distributor in the Kingdom of Saudi Arabia.

### 2.2. Cell Culture and Treatment

Human hepatoma-derived Huh-7 cells obtained from the core facility of KAIMRC in Riyadh were cultured (75 cm^2^ flasks, Corning Glassworks, Corning, NY, USA) in Dulbecco’s modified Eagle’s medium (DMEM) containing low glucose supplemented with L-glutamine and 1% streptomycin/penicillin mixture and 10% fetal bovine serum (FBS). For experiments, cells from 70% to 80% confluent flasks were seeded on 6-well or 12-well plates.

Huh-7 cells were treated with 0.2 mM of palmitate, oleate or a combination of both complexed with 0.25% BSA for 17 h in 10% FBS [27]. Cells were replaced in low glucose supplemented serum-free media for another 4 h and then treated with or without 250 nM bovine insulin for 15 min. In some experiments, Huh-7 cells were treated with 10 µg/mL of tunicamycin to induce ER stress for 6 h, prior to a 15 min acute insulin challenge. Glucose uptake was determined using 10 µM of 2-NBD glucose for 1 h, as described previously [28]. Fluorescence was measured using a Synergy H1 multimode microplate reader from BioTek (Winooski, VT, USA).

To prevent FFA uptake, Huh-7 cells were pre-treated for 6 h with 100 µM of CD36 inhibitor sulfo-*N*-succinimidyl oleate sodium (SSO) [29,30,31] and then incubated for another 17 h with 0.2 mM of palmitate or oleate or 10 µg/mL of tunicamycin in the presence or absence of 100 µM of SSO.

### 2.3. Western Blotting

For the detection of proteins in Huh-7 cells, cells were lysed with RIPA buffer and were separated on 4%–20% Mini-PROTEAN TGX precast protein gels (catalog #4561096) from BioRad (Hercules, CA, USA). Separated proteins were transferred to a PVDF membrane, blocked with 50 mM Tris, pH 7.6, 150 mM NaCl, 0.5% Tween 20 and 5% milk (TBS plus Tween 20) and probed with different primary anti-human antibodies (1:1000 dilution) overnight at 4 °C, followed by incubation with horseradish peroxidase conjugated secondary anti-rabbit/mouse IgG antibodies for 1 h at room temperature. The blots were developed with the Clarity Western ECL substrate (catalog #1705060) from BioRad (Hercules, CA, USA). The results were photographed with the ChemiDoc MP Imaging System from BioRad (Hercules, CA, USA).

### 2.4. mRNA Analysis via Quantitative Real Time PCR

Total RNA from tissues was isolated using TRIzol^TM^ as per the manufacturer’s instructions. The purity of RNA was assessed based on the *A_260_*/*A_280_* ratio. RNA preparations with *A_260_*/*A_280_* ratios more than 1.7 were used for cDNA synthesis. The first-strand cDNA was synthesized using a high-capacity cDNA reverse transcription kit. Each quantitative PCR was carried out in a volume of 20 µL, consisting of 5 µL of cDNA sample (1:100 dilution of the first-strand cDNA sample) and 15 µL of SYBR Green I PCR master mix solution containing 1× PCR buffer. The PCR was carried out by incubating the reaction mixture first for 10 min at 95 °C, followed by 40 cycles of 15 s incubations at 95 °C and 1 min at 60 °C in a QuantStudio™ 6 Flex Real-Time PCR (Applied Biosystems, Waltham, MA, USA). Data were analyzed using the ΔΔC_T_ method, according to the manufacturer’s instructions, and presented as arbitrary units that were normalized to the expression of 18 sRNA. The primers (Table 1) were designed using Primer Express 3.0 (Applied Biosystems).

### 2.5. Oil Red O Staining

The accumulation of fatty acids was detected by staining Huh-7 cells with Oil Red O (catalog #154-02072) from Wako (Richmond, VA, USA). Huh-7 cells were cultured on coverslips in 12-well plates and treated as described above. Cells were washed with phosphate buffered saline (PBS) and fixed in 10% formalin. After washing with PBS, Oil Red O was added. Coverslips containing the cells were transferred onto the slides and mounted using aqueous mounting media. Cells were observed under brightfield (EVOS FL Cell Imaging System, Thermo Fisher Scientific), fluorescence (using FITC and DAPI Fluo channels) and phase contrast (3D Cell Explorer, NanoLive) microscopy and photographs were taken.

### 2.6. Statistical Analysis

All data are presented as the mean ± S.D. The mean values of each group were analyzed via Student’s *t* test using GraphPad Prism software (version 5.0; GraphPad, San Diego, CA, USA). The results with *p* < 0.05 were considered statistically significant.

## 3. Results

### 3.1. Induction of ER Stress by Tunicamycin in Huh-7 Cells Impairs Insulin Signaling through a Reduction in ERK Phosphorylation

**Figure 1:**
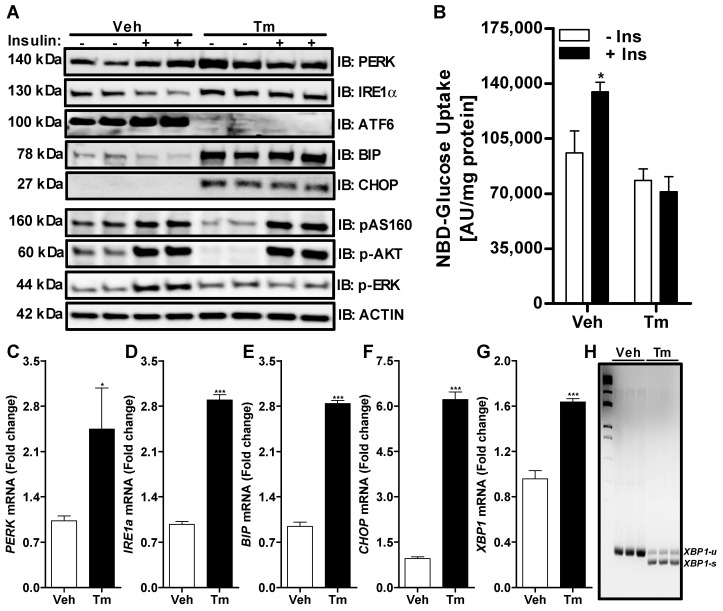
Tunicamycin induced ER stress alters insulin signaling in Huh-7 cells. Huh-7 cells were treated with either DMSO (Veh) or 10 mg/mL of tunicamycin (Tm) for 6 h prior to a 15 min acute challenge with 250 nM of bovine insulin. Cells were used to measure the expression of ER stress and downstream insulin signaling pathway proteins by Western blotting (Panel **A**). In a separate experiment, cells were used to determine the uptake of 2-NBD glucose for 1 h in triplicate after the insulin challenge (Panel **B**). Cells were also used for the extraction of total RNA to measure the expression of various ER stress genes by qRT-PCR (Panels **C**–**G**). Unspliced (u) and spliced (s) forms of *XBP1* mRNA were measured by RT-PCR (Panel **H**). Values were plotted as mean ± SD. *p* values were calculated using Student’s *t* test. *, *p* < 0.05; ***, *p* < 0.001.

ER stress is considered an important event in insulin resistance; therefore, we investigated the ability of tunicamycin, a known ER stress inducer, to regulate insulin signaling in Huh-7 cells. We treated Huh-7 cells with 10 µg/mL of tunicamycin for 6 h, prior to a 15 min acute insulin challenge. Treatment with tunicamycin increased both the protein (Figure 1A) and mRNA (Figure 1C–G) levels of ER stress markers. Tunicamycin also cleaved the ATF6 protein (Figure 1A), which is activated upon cleavage and moves to the nucleus to increase expression of other chaperone genes (Figure 1A,C–G). We also observed an increase in the splicing of *XBP1* mRNA in the presence of tunicamycin compared to DMSO (Veh)-treated cells (Figure 1H). These data confirm the activation of ER stress upon tunicamycin treatment. 

Next, we looked at the effect of ER stress on insulin signaling. In the absence of tunicamycin, an acute insulin challenge induced a significant increase in the phosphorylation of the AKT substrate of 160 kDA (AS160), AKT and ERK (Figure 1A). The increase in the phosphorylation of these proteins by insulin treatment was accompanied by an increase in the uptake of glucose by ~40% in the DMSO-treated cells (Figure 1B). We observed a decrease in the basal phosphorylation levels of AS160 and AKT upon tunicamycin treatment without insulin challenge (Figure 1A). When the tunicamycin-treated cells were challenged with insulin, the phosphorylation of these proteins increased to the same levels as insulin-challenged vehicle treated cells (Figure 1A). However, insulin was unable to increase the phosphorylation of ERK in tunicamycin-treated cells (Figure 1A). The increase in phosphorylation of AS160 and AKT by insulin in the tunicamycin-treated Huh-7 cells failed to increase the uptake of glucose as seen in the vehicle treated cells (Figure 1B), suggesting that AS160 and AKT phosphorylation may not be directly involved in the tunicamycin-induced insulin resistance. Overall, these results demonstrate that the activation of ER stress by tunicamycin reduces insulin-mediated ERK phosphorylation and thereby affects glucose uptake.

### 3.2. Palmitate Induces ER Stress and Reduces ERK Phosphorylation That Affects Insulin Dependent Glucose Uptake in Huh-7 Cells

**Figure 2:**
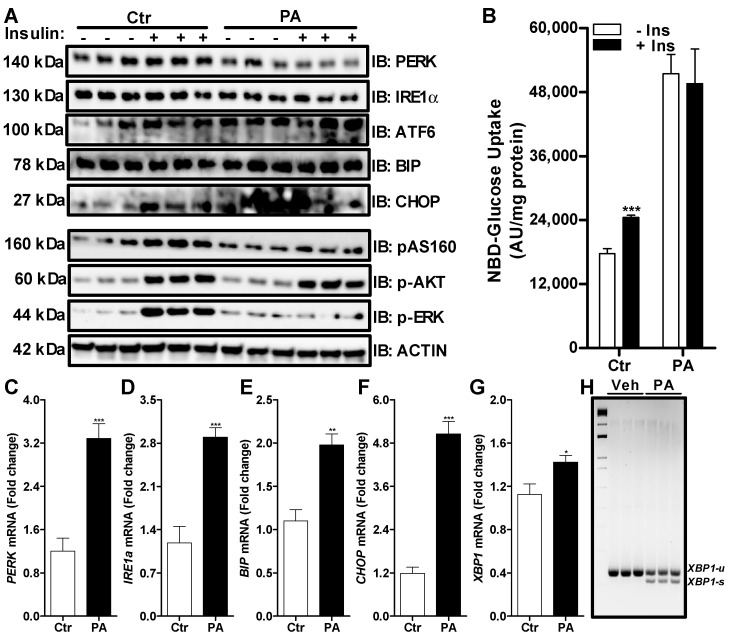
Palmitate induces both an alteration in ER stress and insulin signaling in Huh-7 cells. Huh-7 cells were treated without (Ctr) or with 0.2 mM of palmitate (PA) for 16 h prior to a 15 min challenge with 250 nM of bovine insulin. Cells were used to measure the expression of different ER stress and downstream insulin signaling pathway proteins by Western blotting (Panel **A**). In a separate experiment, cells were used to determine the uptake of 2-NBD glucose for 1 h in triplicate after the insulin challenge (Panel **B**). Cells were also used for the extraction of total RNA to measure the expression of various ER stress genes by qRT-PCR (Panels **C**–**G**). Unspliced (u) and spliced (s) forms of *XBP1* mRNA were measured by RT-PCR (Panel **H**). Values were plotted as mean ± SD. *p* values were calculated using Student’s *t* test. *, *p* < 0.05; **, *p* < 0.01; ***, *p* < 0.001.

To study fatty acid-induced ER stress, we treated Huh-7 cells with palmitate. At a 0.2 mM concentration of palmitate, we did not see any significant change in the protein levels of PERK, IRE1α, ATF6, BIP or CHOP (Figure 2A). However, there was a significant increase in their mRNA levels after the treatment with palmitate (Figure 2C–G). We also noticed an increase in the splicing of *XBP1* mRNA in the presence of palmitate compared to control cells (Figure 2H). These data demonstrate that similarly to tunicamycin, palmitate induces ER stress markers, including the splicing of *XPB1* mRNA.

**Figure 3:**
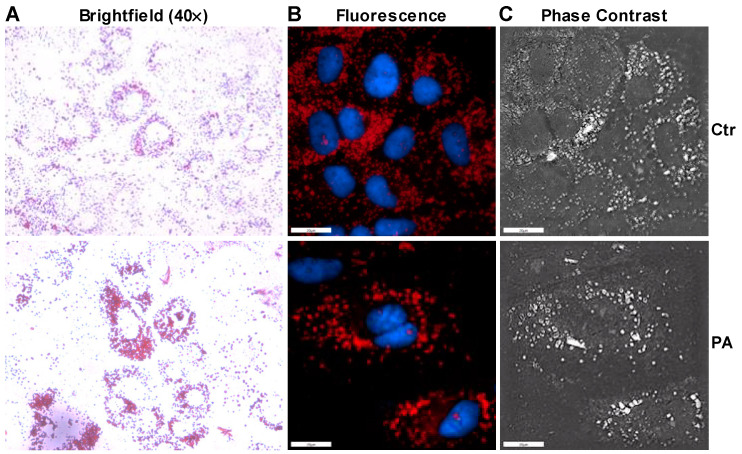
Palmitate induces morphological changes in Huh-7 cells. Huh-7 cells were treated without (Ctr) or with 0.2 mM of palmitate (PA) for 16 h prior to staining them with Oil Red O. Cells were used to determine the morphological changes and accumulation of lipid droplets using the brightfield (Panel **A**), fluorescence (Panel **B**), and phase contrast (Panel **C**) microscopy. Representative photographs of each condition is shown above in the figure.

As shown previously, insulin induced an increase in the phosphorylation of AS160, AKT and ERK in the absence of palmitate treatment in control cells (Figure 2A). This increase in insulin signaling resulted in an increase in the uptake of glucose by 38% in the control cells (Figure 2B). Similarly to tunicamycin, insulin was able to increase the phosphorylation of AKT in the palmitate-treated Huh-7 cells (Figure 2A). However, phosphorylation of AS160 was blunted in the presence of palmitate after the treatment with insulin as compared to insulin-treated control cells (Figure 2A). Again, insulin was unable to increase phosphorylation ERK in the palmitate-treated Huh-7 cells (Figure 2A). We observed a significant increase in the uptake of glucose in the absence of insulin after the treatment of Huh-7 cells with palmitate and this uptake was not further increased by the addition of insulin (Figure 2B). Next, we performed Oil Red O staining to measure the accumulation of lipids in the cells. Brightfield (Figure 3A), fluorescence (Figure 3B) and phase contrast (Figure 3C) microscopy showed that palmitate-treated cells accumulated more lipids compared to control cells. Furthermore, we noticed that cells treated with palmitate looked morphologically different than control cells (Figure 3A). These combined results suggest that palmitate leads to the accumulation of lipids and induces ER stress that alters insulin signaling via ERK phosphorylation.

### 3.3. Oleate Prevents Palmitate-Induced ER Stress without Affecting Insulin Signaling in Huh-7 Cells

**Figure 4:**
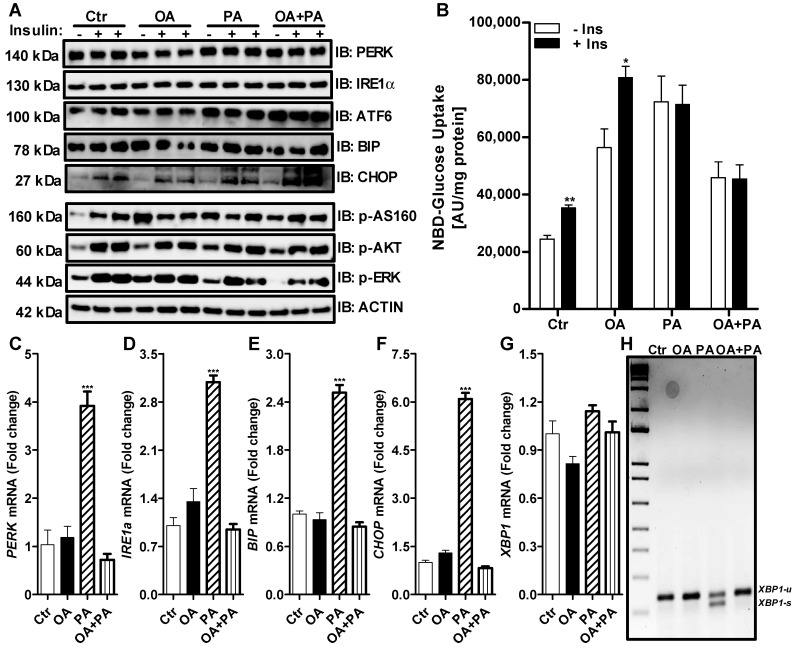
Oleate prevents palmitate induced alteration in ER stress but not ERK phosphorylation in Huh-7 cells. Huh-7 cells were treated without (Ctr) or with 0.2 mM of oleate (OA), palmitate (PA) or a combination of both (OA+PA) for 16 h prior to a 15 min challenge with 250 nM of bovine insulin. Cells were used to measure the expression of different ER stress and downstream insulin signaling pathway proteins by Western blotting (Panel **A**). In a separate experiment, cells were used to determine the uptake of 2-NBD glucose for 1 h in triplicate after the insulin challenge (Panel **B**). Cells were also used for the extraction of total RNA to measure the expression of various ER stress genes by qRT-PCR (Panels **C**–**G**). Unspliced (u) and spliced (s) forms of *XBP1* mRNA were measured by RT-PCR (Panel **H**). Values were plotted as mean ± SD. *p* values were calculated using Student’s *t* test. *, *p* < 0.05; **, *p* < 0.01; ***, *p* < 0.001.

To evaluate the differential effects of palmitate and oleate on ER stress in liver cells, we examined their effects on the expression of the ER stress markers in Huh-7 cells. As seen before (Figure 2), treatment with palmitate significantly increased the expression of ER stress genes compared to control-treated cells (Figure 4C–H). Unlike tunicamycin and palmitate (Figure 1 and Figure 2), 0.2 mM of oleate did not induce the expression of ER stress markers (Figure 4A,C–G). Oleate was also unable to induce the splicing of *XBP1* mRNA (Figure 4H). On the other hand, palmitate-induced ER stress markers and *XBP1* splicing were prevented in the presence of oleate (Figure 4C–H). These results suggest that oleate prevents the palmitate-induced expression of ER stress genes in Huh-7 cells.

**Figure 5:**
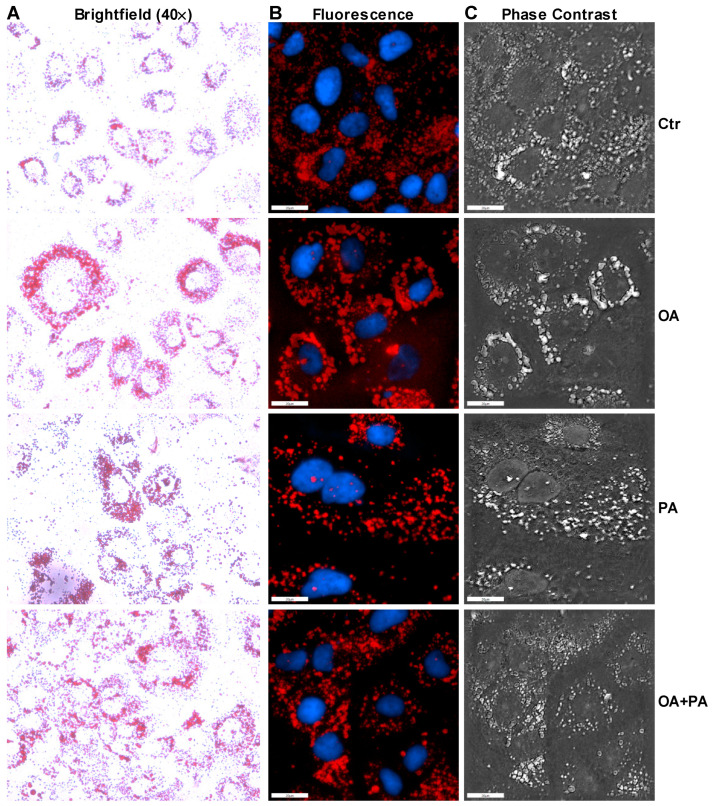
Oleate prevents palmitate induced morphological changes in Huh-7 cells. Huh-7 cells were treated without (Ctr) or with 0.2 mM of oleate (OA), palmitate (OP) or a combination of both (OA+PA) for 16 h prior to staining them with Oil Red O. Cells were used to determine the morphological changes and accumulation of lipids using the brightfield (Panel **A**), fluorescence (Panel **B**), and phase contrast (Panel **C**) microscopy. Representative photographs of each condition is shown above in the figure.

As observed previously, the treatment of control cells (Ctr) with insulin resulted in an increase in the phosphorylation of AS160, AKT and ERK (Figure 4A), which led to an increase of 45% in the uptake of glucose by these cells (Figure 4B). Similar results were obtained when cells were treated with oleate (Figure 4A,B). Again, palmitate treatment resulted in reduced ERK phosphorylation after insulin challenge compared to control- or oleate-treated cells, without any significant change in AS160 or AKT phosphorylation (Figure 4A). This reduction of ERK phosphorylation by palmitate was accompanied by reduced glucose uptake by Huh-7 cells after insulin treatment (Figure 4B). Interestingly, incubation of Huh-7 cells with oleate in combination with palmitate prevented ER stress-related gene expression (Figure 4C–G) but was unable to reverse palmitate-induced insulin resistance, as was depicted by reduced ERK phosphorylation (Figure 4A), and the lessened glucose uptake (Figure 4B) after insulin treatment in these cells. We noticed that basal glucose levels in Huh-7 cells treated with oleate, similarly to palmitate, were significantly higher than the control cells (Figure 4B). Huh-7 cells treated with oleate resulted in the increased accumulation of lipids (Figure 5A, OA) compared to control cells (Figure 5A, Ctr). We also noticed increased basal phosphorylation of AS160, AKT and ERK in oleate-treated cells compared to control cells without insulin treatment (Figure 4A). These combined data suggest that the presence of oleate may prevent palmitate-induced ER stress but may not reverse palmitate-induced insulin resistance in Huh-7 cells.

### 3.4. CD36 Inhibitor Prevents Palmitate-Induced XBP1 Splicing in Huh-7 Cells

**Figure 6:**
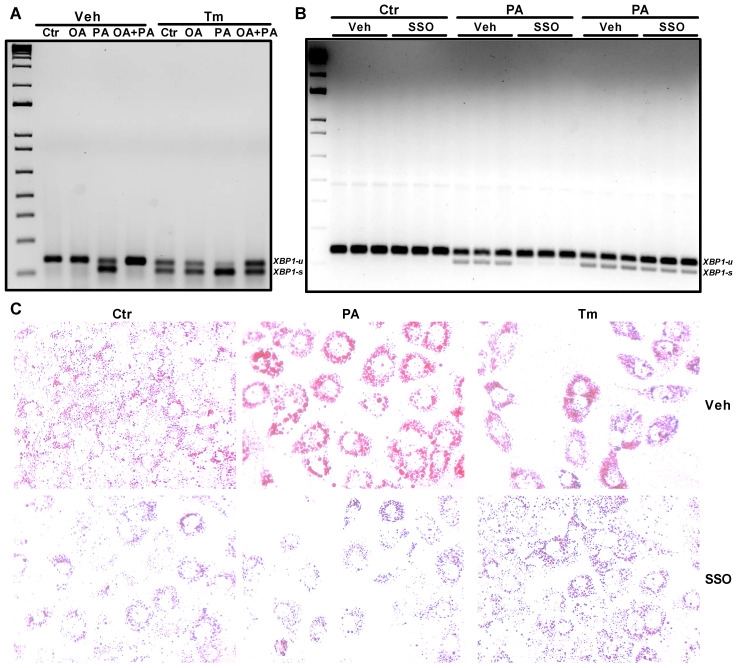
Oleate and SSO prevents palmitate induced and not tunicamycin induced *XBP1* splicing in Huh-7 cells. Huh-7 cells were treated without (Veh) or with 10 mg/mL of tunicamycin for 6 h. Cells were washed and incubated without (Ctr) or with 0.2 mM of oleate (OA), palmitate (PA) or a combination of both (OA+PA) for 16 h (**A**). In a separate experiment, Huh-7 cells were pre-treated with vehicle (Veh) or 100 mM of sulfo-*N*-succinimidyl oleate sodium (SSO) for 6 h. After 6 h, fresh media was added and cells were treated without (Ctr) or with either 0.2 mM palmitate (PA) or 10 mg/mL of tunicamycin (Tm) for another 17 h in the absence (Veh) or presence of 100 mM of SSO. Cells were used for the extraction of total RNA. Unspliced (u) and spliced (s) forms of *XBP1* mRNA were measured by RT-PCR (**B**). Lipid accumulation in SSO treated Huh-7 cells was determined after staining the cells with Oil Red O using the brightfield microscopy (**C**). Representative photographs of each condition is shown above in the figure.

We showed that oleate prevented palmitate-induced ER stress by reducing the expression of ER stress genes, including *XBP1* mRNA splicing in Huh7 cells (Figure 4C–H). To determine whether oleate can also ameliorate tunicamycin-induced *XBP1* splicing, we treated Huh-7 cells with tunicamycin for 6 h and then incubated these cells with either oleate or palmitate or a combination of both. As seen before, the incubation of DMSO (Veh)-treated cells with palmitate increased the splicing of *XBP1* mRNA, which was prevented by the addition of oleate during the incubation period (Figure 6A). Treatment with tunicamycin alone increased the splicing of *XBP1* mRNA, which was further increased in the presence of palmitate, suggesting that the combination of tunicamycin and palmitate had an additive effect on *XBP1* splicing (Figure 6A). Incubation of Huh-7 cells with oleate after the treatment with tunicamycin did not reverse the tunicamycin-induced *XBP1* mRNA splicing (Figure 6A). When tunicamycin-treated cells were incubated with a combination of oleate and palmitate, we observed a reduction in *XBP1* mRNA splicing compared to tunicamycin and palmitate combination-treated cells, suggesting that oleate only reduces palmitate-induced XBP1 splicing. These data also suggest that tunicamycin and palmitate may induce ER stress through the activation of different pathways in Huh-7 cells.

Next, we treated cells with sulfo-*N*-succinimidyl oleate sodium (SSO), which inhibits CD36 transporters to prevent fatty acid uptake by the cells, and looked at the splicing of *XBP1* mRNA (Figure 6B). The incubation of cells with SSO alone did not lead to any *XBP1* mRNA splicing compared to vehicle-treated cells. On the other hand, the incubation of cells with palmitate or tunicamycin resulted in an increase in the splicing of *XBP1* mRNA. Pre-treatment of palmitate- or tunicamycin-treated cells with SSO resulted in the prevention of *XBP1* mRNA splicing only in palmitate-treated cells (Figure 6B). These data suggest that palmitate-mediated ER stress is dependent on fatty acid uptake via CD36, whereas tunicamycin-induced ER stress may be independent of fatty acid uptake and accumulation. To determine whether SSO prevents palmitate- and tunicamycin-induced lipid accumulation, we performed Oil Red O staining in these cells. Our data clearly indicates that the incubation of Huh-7 cells with SSO resulted in the decreased accumulation of lipids in control-, palmitate- and tunicamycin-treated cells (Figure 6C). These data show that, in contrast to palmitate-mediated ER stress, tunicamycin-induced ER stress may be independent of lipid accumulation in Huh-7 cells.

## 4. Discussion

**Figure 7:**
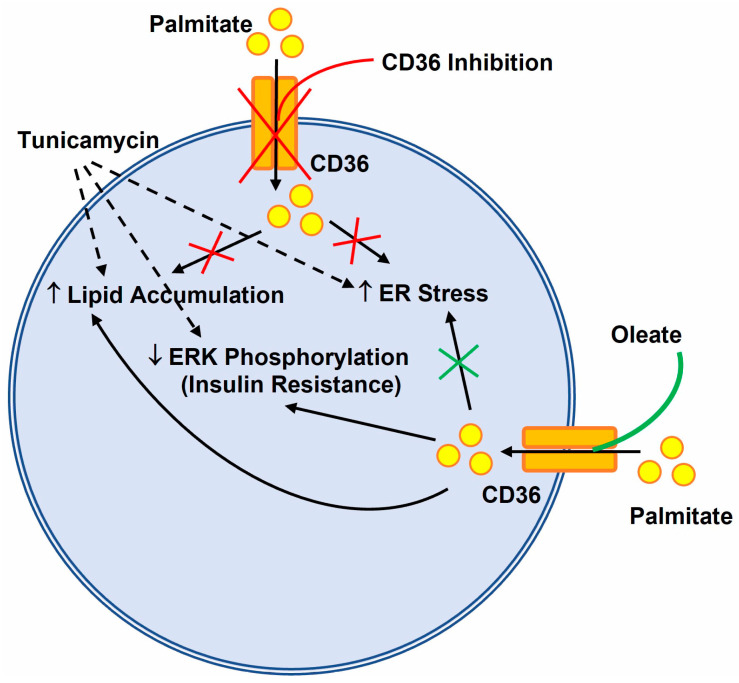
Graphical representation of palmitate induced ER stress and insulin resistance in cells. Palmitate, similar to tunicamycin, elicits an increase in lipid accumulation and ER stress with a concomitant decrease in ERK phosphorylation leading to insulin resistance. Inhibition of CD36 prevents palmitate induced lipid accumulation and ER stress in the cells. Treatment with oleate prevents only palmitate induced ER stress but does not restore ERK phosphorylation or insulin resistance in the cells.

Insulin resistance is characterized by defective insulin signaling through the AKT or ERK signaling pathway [24,32]. Tunicamycin is a naturally occurring antibiotic that induces ER stress in cells by inhibiting protein glycosylation. Our data indicated that treatment of Huh-7 cells with tunicamycin, an agent commonly used to induce the unfolded protein response, increased the expression of ER stress genes (Figure 7), which was accompanied by a marked decrease in the basal levels of AKT phosphorylation. However, contrary to a published report [26], our data showed that tunicamycin-induced ER stress had no effect on insulin-dependent AKT phosphorylation, which was increased to same level as that of vehicle-treated insulin cells. This difference in AKT phosphorylation levels in our study may be because of the shorter tunicamycin treatment (6 h) in contrast to the longer 12–24 h treatment by other investigators [33,34]. It is also possible that different cell lines used in these studies may activate AKT phosphorylation differently after the induction of ER stress by tunicamycin. We also found that the treatment of Huh-7 cells with the ER stressor tunicamycin inhibited the ability of insulin to stimulate ERK phosphorylation and glucose uptake (Figure 7). Again, these results were in contradiction with one of the published reports that showed an increase in ERK phosphorylation after insulin stimulation in tunicamycin-treated cells [26]. In this study, the investigators treated myotube skeletal muscle cells with 5 µg/mL of tunicamycin for only 3 h. The use of a lower concentration of tunicamycin with a shorter incubation time and a different cell line may be the reason for the discrepancy observed in the results between the current and published studies. These combined data suggest that the incubation of varied cell lines with different concentrations of tunicamycin for different durations may produce different outcomes in terms of insulin signaling.

A high level of FFAs in the circulation triggers ER stress and insulin resistance in peripheral tissues such as the liver and muscles [16,35]. Treatment of cells with saturated fatty acid palmitate induces ER stress and the inactivation of insulin signaling [16,34,35,36]. In the current study, Huh-7 cells looked morphologically different compared to control cells at a lower concentration of palmitate (0.2 mM). Similarly to tunicamycin treatment, palmitate resulted in significant changes in the expression of ER stress genes and affected insulin signaling and glucose uptake by the cells (Figure 7). Both tunicamycin and palmitate treatment resulted in a decrease in ERK phosphorylation without any change in AKT phosphorylation upon insulin challenge. A study has shown that palmitate increases the phosphorylation of ribosomal S6 kinase 1 (S6K1) in hepatocytes and inhibition of this phosphorylation leads to a reduction in ER stress-mediated lipotoxicity and insulin resistance induced by palmitate [37].

Our data show that palmitate is lipotoxic for Huh-7 cells and induces ER stress through the activation of *XBP1* mRNA splicing, similarly to tunicamycin. These results were in agreement with the findings obtained in other studies [16,33,34]. Consistent with the published data, our data also indicate that unsaturated fatty acid oleate is not lipotoxic, either alone or in combination with palmitate [13]. Similarly to the findings of a published report [16], oleate was able to prevent palmitate-induced ER stress by mitigating the induction of ER stress markers and completely blocking the splicing of *XBP1* mRNA in the current study. However, this prevention in the induction of ER stress by oleate did not reverse the palmitate-induced effect on ERK phosphorylation and glucose uptake (Figure 7).

The basal glucose levels in Huh-7 cells treated with palmitate or oleate were higher than those of the control cells, which was consistent with a report showing increased basal glucose levels with oleate in isolated adipocytes from rats [38]. This increased basal glucose level by oleate in the isolated adipocytes was not due to any changes in the distribution of glucose transporter type 1 (GLUT1) or GLUT4 in the plasma membrane [38]. This may be due to changes in the lipid composition of the cells that may induce morphological changes in the cell membrane, leading to increased glucose permeability. Since oleate was unable to cause any ER stress in the cells and prevented palmitate-mediated but not tunicamycin-induced ER stress, it may be inferred that lipid accumulation per se may not be the cause of ER stress. It is possible that the uptake of palmitate through the fatty acid transporter CD36 into the cells is necessary to cause ER stress and the presence of oleate may diminish this entry. Treatment of Huh-7 cells with the CD36 inhibitor SSO decreased the accumulation of lipids in the cells and prevented palmitate-induced but not tunicamycin induced *XBP1* mRNA splicing.

In summary, these data indicate that palmitate and tunicamycin induces ER stress and alters insulin signaling through a reduction in ERK phosphorylation. The addition of oleate or SSO prevents palmitate-induced but not tunicamycin-induced ER stress. However, the presence of oleate does not prevent the palmitate-induced reduction in ERK phosphorylation. Our results do not provide any evidence regarding whether ERK phosphorylation contributes to ER stress in Huh-7 cells as shown through the reduction in AMPK levels caused by palmitate in skeletal muscles [16]. Rather we conclude that palmitate does not decrease ERK phosphorylation in Huh-7 cells via induction in ER stress. With the evidence provided above, we believe that ER stress may not be the sole mechanism of palmitate-induced insulin resistance in Huh-7 cells and further studies are warranted to investigate these important observations. Experiments with different concentrations or durations of exposure with palmitate may be helpful in understanding the chronic or acute effect of ER stress in insulin resistance.

## Figures and Tables

**Table 1:** List of primers used for the quantification of ER stress genes.

Gene	Forward Primer	Reverse Primer
*PERK*	5′-CAGAGATTGAGACTGCGTGGC-3′	5′-AAGGAACCGGATCCCACATC-3′
*IRE1a*	5′-TTGGGCGAACAGAGATGTCC-3′	5′-CACAGGGGAGGCGTAGTTTT-3′
*BIP*	5′-CGGGCAAAGATGTCAGGAAAG-3′	5′-CAGATGATACTTCGGGCAGGTC-3′
*CHOP*	5′-ACCAAGGGAGAACCAGGAAACG-3′	5′-CGAGACTAACTGGCTTACCACT-3′
*XBP1*	5′-TTACGAGAGAAAACTCATGGC-3′	5′-CGTAAGACCTGTTGAACCTGGG-3′

## Data Availability

The data presented in this study are available on request from the corresponding author.

## References

[1] Boden G. (1997). Role of fatty acids in the pathogenesis of insulin resistance and NIDDM. Diabetes.

[2] Ertunc M.E., Hotamisligil G.S. (2016). Lipid signaling and lipotoxicity in metaflammation: Indications for metabolic disease pathogenesis and treatment. J. Lipid Res..

[3] Kien C.L., Bunn J.Y., Stevens R., Bain J., Ikayeva O., Crain K., Koves T.R., Muoio D.M. (2014). Dietary intake of palmitate and oleate has broad impact on systemic and tissue lipid profiles in humans. Am. J. Clin. Nutr..

[4] Ricchi M., Odoardi M.R., Carulli L., Anzivino C., Ballestri S., Pinetti A., Fantoni L.I., Marra F., Bertolotti M., Banni S. (2009). Differential effect of oleic and palmitic acid on lipid accumulation and apoptosis in cultured hepatocytes. J. Gastroenterol. Hepatol..

[5] Staiger K., Staiger H., Weigert C., Haas C., Haring H.U., Kellerer M. (2006). Saturated, but not unsaturated, fatty acids induce apoptosis of human coronary artery endothelial cells via nuclear factor-kappaB activation. Diabetes.

[6] El-Assaad W., Buteau J., Peyot M.L., Nolan C., Roduit R., Hardy S., Joly E., Dbaibo G., Rosenberg L., Prentki M. (2003). Saturated fatty acids synergize with elevated glucose to cause pancreatic beta-cell death. Endocrinology.

[7] Karaskov E., Scott C., Zhang L., Teodoro T., Ravazzola M., Volchuk A. (2006). Chronic palmitate but not oleate exposure induces endoplasmic reticulum stress, which may contribute to INS-1 pancreatic beta-cell apoptosis. Endocrinology.

[8] Miller T.A., LeBrasseur N.K., Cote G.M., Trucillo M.P., Pimentel D.R., Ido Y., Ruderman N.B., Sawyer D.B. (2005). Oleate prevents palmitate-induced cytotoxic stress in cardiac myocytes. Biochem. Biophys. Res. Commun..

[9] Wei Y., Wang D., Topczewski F., Pagliassotti M.J. (2006). Saturated fatty acids induce endoplasmic reticulum stress and apoptosis independently of ceramide in liver cells. Am. J. Physiol Endocrinol. Metab..

[10] Listenberger L.L., Han X., Lewis S.E., Cases S., Farese R.V., Ory D.S., Schaffer J.E. (2003). Triglyceride accumulation protects against fatty acid-induced lipotoxicity. Proc. Natl. Acad. Sci. USA.

[11] Soumura M., Kume S., Isshiki K., Takeda N., Araki S., Tanaka Y., Sugimoto T., Chin-Kanasaki M., Nishio Y., Haneda M. (2010). Oleate and eicosapentaenoic acid attenuate palmitate-induced inflammation and apoptosis in renal proximal tubular cell. Biochem. Biophys. Res. Commun..

[12] Thorn K., Bergsten P. (2010). Fatty acid-induced oxidation and triglyceride formation is higher in insulin-producing MIN6 cells exposed to oleate compared to palmitate. J. Cell Biochem..

[13] Peng G., Li L., Liu Y., Pu J., Zhang S., Yu J., Zhao J., Liu P. (2011). Oleate blocks palmitate-induced abnormal lipid distribution, endoplasmic reticulum expansion and stress, and insulin resistance in skeletal muscle. Endocrinology.

[14] Kim D.H., Cho Y.M., Lee K.H., Jeong S.W., Kwon O.J. (2017). Oleate protects macrophages from palmitate-induced apoptosis through the downregulation of CD36 expression. Biochem. Biophys. Res. Commun..

[15] Colvin B.N., Longtine M.S., Chen B., Costa M.L., Nelson D.M. (2017). Oleate attenuates palmitate-induced endoplasmic reticulum stress and apoptosis in placental trophoblasts. Reproduction.

[16] Salvado L., Coll T., Gomez-Foix A.M., Salmeron E., Barroso E., Palomer X., Vazquez-Carrera M. (2013). Oleate prevents saturated-fatty-acid-induced ER stress, inflammation and insulin resistance in skeletal muscle cells through an AMPK-dependent mechanism. Diabetologia.

[17] Flamment M., Kammoun H.L., Hainault I., Ferre P., Foufelle F. (2010). Endoplasmic reticulum stress: A new actor in the development of hepatic steatosis. Curr. Opin. Lipidol..

[18] Nakamura S., Takamura T., Matsuzawa-Nagata N., Takayama H., Misu H., Noda H., Nabemoto S., Kurita S., Ota T., Ando H. (2009). Palmitate induces insulin resistance in H4IIEC3 hepatocytes through reactive oxygen species produced by mitochondria. J. Biol. Chem..

[19] Ruddock M.W., Stein A., Landaker E., Park J., Cooksey R.C., McClain D., Patti M.E. (2008). Saturated fatty acids inhibit hepatic insulin action by modulating insulin receptor expression and post-receptor signalling. J. Biochem..

[20] Bjornholm M., Kawano Y., Lehtihet M., Zierath J.R. (1997). Insulin receptor substrate-1 phosphorylation and phosphatidylinositol 3-kinase activity in skeletal muscle from NIDDM subjects after in vivo insulin stimulation. Diabetes.

[21] Krook A., Bjornholm M., Galuska D., Jiang X.J., Fahlman R., Myers M.G., Wallberg-Henriksson H., Zierath J.R. (2000). Characterization of signal transduction and glucose transport in skeletal muscle from type 2 diabetic patients. Diabetes.

[22] Andreasson K., Galuska D., Thorne A., Sonnenfeld T., Wallberg-Henriksson H. (1991). Decreased insulin-stimulated 3-0-methylglucose transport in in vitro incubated muscle strips from type II diabetic subjects. Acta Physiol. Scand..

[23] Jiang Z.Y., Zhou Q.L., Coleman K.A., Chouinard M., Boese Q., Czech M.P. (2003). Insulin signaling through Akt/protein kinase B analyzed by small interfering RNA-mediated gene silencing. Proc. Natl. Acad. Sci. USA.

[24] Ozaki K.I., Awazu M., Tamiya M., Iwasaki Y., Harada A., Kugisaki S., Tanimura S., Kohno M. (2016). Targeting the ERK signaling pathway as a potential treatment for insulin resistance and type 2 diabetes. Am. J. Physiol. Endocrinol. Metab..

[25] Tang X., Shen H., Chen J., Wang X., Zhang Y., Chen L.L., Rukachaisirikul V., Jiang H.L., Shen X. (2011). Activating transcription factor 6 protects insulin receptor from ER stress-stimulated desensitization via p42/44 ERK pathway. Acta Pharmacol. Sin..

[26] Hwang S.L., Jeong Y.T., Li X., Kim Y.D., Lu Y., Chang Y.C., Lee I.K., Chang H.W. (2013). Inhibitory cross-talk between the AMPK and ERK pathways mediates endoplasmic reticulum stress-induced insulin resistance in skeletal muscle. Br. J. Pharmacol..

[27] Powell D.J., Turban S., Gray A., Hajduch E., Hundal H.S. (2004). Intracellular ceramide synthesis and protein kinase Czeta activation play an essential role in palmitate-induced insulin resistance in rat L6 skeletal muscle cells. Biochem. J..

[28] Yoshioka K., Oh K.B., Saito M., Nemoto Y., Matsuoka H. (1996). Evaluation of 2-[N-(7-nitrobenz-2-oxa-1,3-diazol-4-yl)amino]-2-deoxy-D-glucose, a new fluorescent derivative of glucose, for viability assessment of yeast Candida albicans. Appl. Microbiol. Biotechnol..

[29] Kuda O., Pietka T.A., Demianova Z., Kudova E., Cvacka J., Kopecky J., Abumrad N.A. (2013). Sulfo-N-succinimidyl oleate (SSO) inhibits fatty acid uptake and signaling for intracellular calcium via binding CD36 lysine 164: SSO also inhibits oxidized low density lipoprotein uptake by macrophages. J. Biol. Chem..

[30] Nath A., Li I., Roberts L.R., Chan C. (2015). Elevated free fatty acid uptake via CD36 promotes epithelial-mesenchymal transition in hepatocellular carcinoma. Sci. Rep..

[31] Drury J., Rychahou P.G., He D., Jafari N., Wang C., Lee E.Y., Weiss H.L., Evers B.M., Zaytseva Y.Y. (2020). Inhibition of Fatty Acid Synthase Upregulates Expression of CD36 to Sustain Proliferation of Colorectal Cancer Cells. Front. Oncol..

[32] Huang X., Liu G., Guo J., Su Z. (2018). The PI3K/AKT pathway in obesity and type 2 diabetes. Int. J. Biol. Sci..

[33] Ijuin T., Hosooka T., Takenawa T. (2016). Phosphatidylinositol 3,4,5-Trisphosphate Phosphatase SKIP Links Endoplasmic Reticulum Stress in Skeletal Muscle to Insulin Resistance. Mol. Cell Biol..

[34] Hage Hassan R., Hainault I., Vilquin J.T., Samama C., Lasnier F., Ferre P., Foufelle F., Hajduch E. (2012). Endoplasmic reticulum stress does not mediate palmitate-induced insulin resistance in mouse and human muscle cells. Diabetologia.

[35] Schmitz-Peiffer C., Craig D.L., Biden T.J. (1999). Ceramide generation is sufficient to account for the inhibition of the insulin-stimulated PKB pathway in C2C12 skeletal muscle cells pretreated with palmitate. J. Biol. Chem..

[36] Achard C.S., Laybutt D.R. (2012). Lipid-induced endoplasmic reticulum stress in liver cells results in two distinct outcomes: Adaptation with enhanced insulin signaling or insulin resistance. Endocrinology.

[37] Pardo V., Gonzalez-Rodriguez A., Muntane J., Kozma S.C., Valverde A.M. (2015). Role of hepatocyte S6K1 in palmitic acid-induced endoplasmic reticulum stress, lipotoxicity, insulin resistance and in oleic acid-induced protection. Food Chem. Toxicol..

[38] Murer E., Boden G., Gyda M., Deluca F. (1992). Effects of oleate and insulin on glucose uptake, oxidation, and glucose transporter proteins in rat adipocytes. Diabetes.

